# Meta-Qtest: meta-analysis of quadratic test for rare variants

**DOI:** 10.1186/s12920-019-0516-5

**Published:** 2019-07-11

**Authors:** Jieun Ka, Jaehoon Lee, Yongkang Kim, Bermseok Oh, T2D-GENES Consortium, Taesung Park

**Affiliations:** 1 Department of Statistics, Seoul National University, Seoul, South Korea; 2 Department of Biochemistry and Molecular Biology, School of Medicine, Kyung Hee University, Seoul, South Korea; 3 Interdisciplinary program in Bioinformatics, Seoul National University, Seoul, South Korea

**Keywords:** Meta-analysis, Rare variant analysis, Exome sequencing, Meta-Qtest

## Abstract

**Background:**

In genome-wide association studies (GWASs), meta-analysis has been widely used to improve statistical power by combining the results of different studies. Meta-analysis can detect phenotype associated variants that are failed to be detected in single studies. Especially, in biomedical sciences, meta-analysis is often necessary not only for improving statistical power, but also for reducing unavoidable limitation in data collection. As next-generation sequencing (NGS) technology has been developed, meta-analysis of rare variants is proceeding briskly along with meta-analysis of common variants in GWASs. However, meta-analysis on a single variant that is commonly used in common variant association test is improper for rare variants. A sparse signal of rare variant undermines the association signal and its large number causes multiple testing problem. To over-come these problems, we propose a meta-analysis method at the gene-level rather than variant level.

**Results:**

Among many methods that have been developed, we used the unified quadratic tests (Q-tests); Q-test is more powerful than or as powerful as other tests such as Sequence Kernel Association Tests (SKAT). Since there are three different versions of Q-test (QTest1, QTest2, QTest3), each assumes different relationships among multiple rare variants, we extended them into meta-study accordingly. For meta-analysis, we consider two types of approaches, the one is to combine regression coefficients and the other is to combine test statistics from each single study. We extend the Q-test for meta-analysis, proposing Meta Quadratic Test (Meta-Qtest). Meta Q-test avoids the limitations of MetaSKAT. It does not only consider genetic heterogeneity among studies as MetaSKAT but also reflects diverse real situations; since we extend three different Q-tests into meta-analysis respectively, flexible Meta Q-test suggests way to deal with gene-level rare variant meta-analysis efficiently From the results of real data analysis of blood pressure trait, our meta-analysis could successfully discovered genes, KCNA5 and CABIN1 that are already well known for relevance with hypertension disease and they are not detected in MetaSKAT.

**Conclusion:**

As exemplified by an application to T2D Genes projects data set, Meta-Qtest more effectively identified genes associated with hypertension disease than MetaSKAT did.

## Background

Genome-wide association studies (GWASs) have identified many loci that contributed to human complex traits. As genotyping technologies such as next-generation sequencing (NGS) technology evolve, we have been able to gain larger data and more accurate information on human genetics. Discovery of rare variants is one of the most valuable crops of the NGS technologies [1]. The subject of analysis naturally went over from common variants to relatively less studied rare variants, because GWASs on common variants could not entirely explain genetic-heritability, only explains small portion of expected heritability. Such phenomenon, known as “missing heritability”, posed the necessity of analyzing rare variant in human disease with a belief that rare variants play an important role in association study [2].

Persisting on same methods in common variant analyses is not appropriate for dealing with the rare variants [3]. Due to the fact that only few people share rare variants, we need a larger sample size than in common variant association test. Small sample size could markedly lower the power of a statistical test. Besides, if each variant effect is weak then single variant analysis has lower power to detect true weak signal. Therefore, in such situations instead of single variant association test, gene level test that handles multiple variants in a gene could be helpful in strengthening the signals by considering several weak ones at a time [4]. In addition to the benefit of increasing the power, gene-based multi-marker test mitigate the burden of multiple testing correction and easily interpret biological functional meaning of detected genes from the result of test. For these reasons, gene-based test is often used for rare variants analysis.

Over the past few years, various statistical methods for gene-level rare variant association test are developed. From collapsing based methods such as Combined Multivariate and Collapsing (CMC) and variable thresholds test (VT test) to variance component tests such as C-alpha and Sequence Kernel Association Tests (SKAT or SKAT-O), each method performs well in different situations [5–9]. CMC method is the one of most representative burden type tests. It unifies collapsing technique and multivariate t-test, Hotelling’s T-test. Based on variants’ minor allele frequency (MAF), variants are divided into several sub-groups, then their genotype values are summarized in 0 or 1. With collapsed genotype values, Hotelling’s T-test is conducted. Likewise, CMC or VT test is also well used burden test, but it is adaptive burden test. Compared to regular burden test like CMC, VT test allows flexible MAF threshold. Since appropriate threshold can impact on power, VT test can increase the power by choosing the optimal threshold that maximizes test statistic [6]. CMC and VT test are powerful under the assumptions of high proportion of causal variants and same direction of their effects on certain disease; most single-nucleotide polymorphisms (SNPs) in a gene are causal and they are all protective or deleterious. When a small number of SNPs are causal and some of causal are protective and others are deleterious in a gene, burden test loses the power, and C-alpha or SKAT outperform them. Both C-alpha and SKAT are variance component test that test the variance components instead of means. C-alpha test is designed for case-control studies without covariates. Under null hypothesis that says that no variants are associated with a phenotype, for case-control data, the distribution of allele counts follows binomial distribution. It compares the observed variance of counts with expected variance. The test statistic for C-alpha includes squared terms that are observed sample variances, thus C-alpha is robust even in the presence of different directions of variants’ effects (because the signs of effects are canceled out and only their effect sizes remain in the test statistic). Despite of the advantage as noted earlier, C-alpha has some disadvantages too. *P* value is computed using permutation that requires intensive computation and covariate adjust is not available. The method proposed to solve these problems is SKAT. SKAT is variance component score test implemented in a regression framework. To test the null hypothesis of zero regression coefficients effect sizes of genetic variants in a gene, SKAT assumes that each regression coefficient follows arbitrary distribution with zero mean and the variance, product of weight and variance component. Then, testing original null hypothesis becomes the same as testing whether each variance component is zero or not. Variance component score statistic is employed in this process. Since SKAT is derived from regression model, it can include covariate terms easily. *P* value is calculated analytically and diverse kernel functions that can explain genetic similarity between individuals are introduced. Furthermore, optimal version of SKAT, SKAT-O is proposed to achieve robust power regardless of directions of variants. SKAT-O is combination of burden test and SKAT, weighted average of burden and SKAT. SKAT-O searches the optimal weight that is the weight obtaining the minimum *p* value of test statistics. However, even though robust power is substantially attained by SKAT-O test, there is still no uniformly most powerful test in all situations [10].

Gene-level Q-test is another powerful rare variant association test [11]. It also uses classical multiple linear regression as well as SKAT, but it takes Wald test based on an eigenvalue decomposition of regression coefficients. Quadratic form of test statistics in Q-test is efficiently implemented in diverse scenarios which embrace various cases in the relation of SNPs in a gene. First, Q-test considers proportion and effect sizes of causal rare variants. Second, it considers the direction of causal variant effects. Finally, it can be used even in the presence of rare variants with common variants, together; this is the major advantage of Q-test. In other words, Q-test could achieve robust power in any case, and its exceeding power was verified in enormous simulation studies.

Meta-analysis is a popular approach to increase the power in GWASs [12]. Aggregated summary from diverse studies recovers as much information as individual-level data but without any exertion of pooling early stage data sets. In this respect, meta-study has an advantage of increasing the sample size and preserving computational efficiency [13]. Meta-analysis is sometimes essential in inevitable circumstances where individual-level data cannot be distributed although quickly advancing NGS technologies allow us to have sequencing data at smaller cost than before, producing data still requires considerable time and money. Not only because of this, but also because of releasing personal data to public is a sensitive issue, not all individual-level datasets are shared. Therefore, meta-analysis, which requires results only, becomes exceptionally useful.

The recently proposed MetaSKAT is extended SKAT for meta-analysis for gene-level rare variants. MetaSKAT aggregates the score statistics of each variants in a gene came from SKAT. Depending on the assumption of genetic effect, homogeneity or heterogeneity of genetic effects across studies, it aggregates the summary score statistics. When genetic effects are homogeneous, summary statistics are combined across the studies first and then combined across the variants in a gene, but when genetic effects are heterogeneous, the combining order is reversed. Optimal MetaSKAT that is weighted sum of test statistic of MetaSKAT and its burden for meta-analysis is also proposed to embrace the merits of burden and non-burden test together. However, simulation results of MetaSKAT show that type I error rates are somewhat uncontrolled. There is another limitation of MetaSKAT. When cohort specific genes are detected by MetaSKAT, it does not report that which cohort is highly associated with a phenotype [14].

In this paper, we extend the Q-test for meta-analysis, proposing Meta Quadratic Test (Meta-Qtest). Meta Q-test avoids the limitations of MetaSKAT. It does not only consider genetic heterogeneity among studies as MetaSKAT but also reflects diverse real situations; since we extend three different Q-tests into meta-analysis respectively, flexible Meta Q-test suggests way to deal with gene-level rare variant meta-analysis efficiently.

## Materials and methods

### Q-test for single study

Q-test is established in a multiple linear regression framework for a quantitative trait.

1 $$ {\mathrm{y}}_{ki}={\beta}_{k0}+\sum \limits_{j=1}^m{\beta}_{kj}{S}_{kj i}+\gamma {Z}_{ki}+{\epsilon}_{ki},\kern1.25em {\epsilon}_{ki}\sim N\left(0,{\sigma}_k^2\right), $$

where y_*ki*_ is the phenotype of the ith individual in the kth study, *S*_*kji*_ is the genotype value coded 0, 1 or 2 under an additive genotype model (dominant or recessive model is also applicable), *β*_*k*_ = (*β*_*k*0_, ⋯, *β*_*km*_)^,^ is the vector of regression coefficients for genetic effects of m SNPs, *Z*_*ki*_ is the covariate value, and *γ* is the corresponding vector of regression coefficients. After fitting model, using the estimated regression coefficients, *β*_*k*_, QTest1 creates the new variable, pooled coefficients, *β*_*pooled*, *k*_. Then, the null hypothesis of interest is to test whether pooled genetic effect exists or not:

$$ {H}_0:{\beta}_{pooled,k}=0. $$

However, for QTest2 we test the vector of regression coefficients, *β*_*k*_, rather than pooled coefficient to allow for considering bidirectional variants effect. Corresponding null hypothesis is

$$ {H}_0:{\beta}_k=\mathbf{0}\ \mathrm{or}\ {\beta}_{k0}={\beta}_{k1}=\dots ={\beta}_{km}=0. $$

To test the hypothesis, Q-test constructs a Wald-type statistic which has the form of quadratic statistics. Depending on the different assumptions needed to collapse the estimates of effects size parameters, there are three versions of Q-test: QTest1, QTest2, and QTest3.

#### Burden Test: Quadratic Test1 (QTest_1_)

QTest_1_ is a burden type of test. A basic assumption is that all of SNPs in a gene have the same direction of effects on the phenotype. That is, we assume that all variants within a region are all deleterious or protective. If the assumption is true, the power of the burden test becomes higher by aggregated effects of each variant. When combining variants, Qtest_1_ uses the inverse variance weighting method that gives more weight to SNPs that have smaller variances,

$$ {\alpha}_{kj}=\frac{1/\mathit{\operatorname{var}}\left({\hat{\beta}}_{kj}\right)}{\sum \limits_{j=1}^m\left(1/\mathit{\operatorname{var}}\left({\hat{\beta}}_{kj}\right)\right)}. $$

Also, like SKAT, QTest_1_ introduces MAF based weight,${\mathrm{w}}_{kj}=1/\sqrt{MAF_j\left(1-{MAF}_j\right)}$, proposed by Madsen and Browning [15]. By using the MAF based weight, our research focus on the rare SNPs is justified (rarer variant has higher probability of being causal variant). With these two different weights, aggregated effects are expressed as ${\hat{\beta}}_{pooled,k}={\alpha}_k^T{W}_k{\hat{\beta}}_k,\kern0.5em \mathrm{where}\ {\alpha}_k$ is the vector of each variant’s inverse variance weight and *W*_*k*_ is the diagonal matrix of each variant’s MAF based weight,

$$ {W}_k=\left[\begin{array}{c}{w}_{k1}\ \\ {}\vdots \\ {}0\end{array}\begin{array}{c}\cdots \\ {}\ddots \\ {}\cdots \end{array}\begin{array}{c}0\\ {}\vdots \\ {}{w}_{km}\ \end{array}\right]. $$

${\hat{\beta}}_k$is the vector of estimated coefficients of variants. In the ordinal regression framework, ${\hat{\beta}}_{pooled,k}$ follows a normal distribution with zero mean and ${\alpha}_k^T{W}_k\mathit{\operatorname{var}}\left({\hat{\beta}}_k\right){W}_k{\alpha}_k$ variance. Based on the distribution of ${\hat{\beta}}_{pooled,k}$, the Wald type of statistics QTest_1_ is given by

$$ {\mathrm{Q}}_1={\left({\alpha}_k^T{W}_k\mathit{\operatorname{var}}\left({\hat{\beta}}_k\right){W}_k{\alpha}_k\right)}^{-1}{{\hat{\beta}}_{pooled,k}}^2\sim {\upchi}_1^2. $$

#### Non-burden test: quadratic Test2 (QTest_2_)

In case of mixed variants in direction of effect, Qtest_1_ would work poorly. Thus, QTest_2_ assumes that some of variants are protective, and the others are negative. Instead of aggregating the effects of variants, it directly constructs Wald statistics for ${\hat{\beta}}_k$,

$$ {\mathrm{Q}}_2^{Wald}={\hat{\beta}}_k^T\mathit{\operatorname{var}}{\left({\hat{\beta}}_k\right)}^{-1}{\hat{\beta}}_k\sim {\upchi}_m^2, $$

where m is the number of variants in a gene. However, the number of rare SNPs in a gene is usually large, so a large degree of freedom lowers the power. Thus, QTest2 proposes a gamma method that could lower the degree of freedom [16].

$$ {\mathrm{Q}}_2^{Wald}={\hat{\beta}}_k^T\mathit{\operatorname{var}}{\left({\hat{\beta}}_k\right)}^{-1}{\hat{\beta}}_k={\hat{\beta}}_k^T{U}_k{\Lambda}^{-1}{U}_k^T{\hat{\beta}}_k\sim {\upchi}_m^2, $$

$$ \mathrm{where},\kern0.5em \mathit{\operatorname{var}}\left({\hat{\beta}}_k\right)=U\Lambda {U}^T\mathrm{and}\ \Lambda =\mathit{\operatorname{diag}}\left({\lambda}_k\right). $$

$$ {p}_{kj}=2\left(1-\Phi \left({u}_{kj}^T{\hat{\beta}}_k/\sqrt{\lambda_{kj}}\right)\right), $$

$$ \mathrm{where}\kern1.75em {u}_{kj}={j}^{th}\ \mathrm{column}\ \mathrm{of}\ {U}_k. $$

The final form of Qtest2 statistics is

$$ {\mathrm{Q}}_2={\sum}_{j=1}^m{G}_{a,1}^{-1}\left(1-{p}_{kj}\right)\sim 1/2{\upchi}_{2 ma}^2. $$

#### Optimal test (unified test): quadratic Test3 (QTest_3_)

Qtest_3_ is an optimal method, weighted average of burden type (Qtest_1_) and non-burden type (Qtest_2_) statistics. To combine two statistics that follow chi square distributions with different degrees of freedoms, there is a step for making Q_2_ has the same degree of freedom as Q_1_ (df = 1). To summarize the steps, first we define the new parameter, ${\hat{\beta}}_k^{\ast }$ that is independent with ${\hat{\beta}}_{pooled,k}$, and based on the new defined ${\hat{\beta}}_k^{\ast }$, we can get the Q_2_ statistics, ${Q}_{2\mid 1}^{\ast }$. Next step is to use a gamma method to make ${Q}_{2\mid 1}^{\ast }$ has 1 degree of freedom (*Q*_2 ∣ 1_). Using final two statistics, Q_1_ and *Q*_2 ∣ 1_, we can get the optimal Qtest_3_ statistics through a grid search of weight. Final *p* value of Q_3_ is calculated by empirically. Pre-calculated empirical distributions are employed in the final step, so the computational burden is reduced.

Since Qtest_3_ could accommodate both scenarios that assume same or different direction of SNPs in a gene, its result is usually robust. The followings are detailed steps of Qtest_3_.

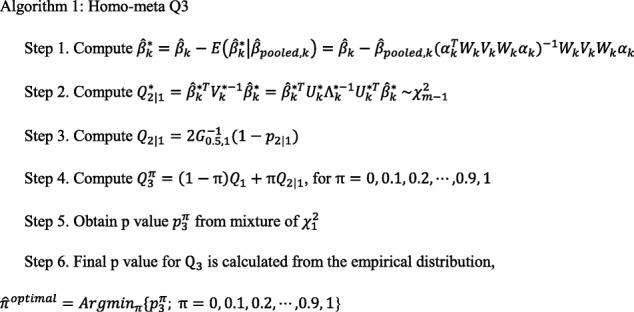



In step 1 in algorithm 1, we compute ${\hat{\beta}}_k^{\ast }$ to make ${\hat{\beta}}_k^{\ast}\perp {\hat{\beta}}_{pooled,k}$. In step 2, we compute ${Q}_{2\mid 1}^{\ast }$, where ${V}_k^{\ast }=\mathit{\operatorname{var}}\left({\hat{\beta}}_k^{\ast}\right)$ and ${U}_k^{\ast }$ consists of eigenvalue vectors of ${V}_k^{\ast }$, and ${\Lambda}_k^{\ast }$ is the diagonal matrix whose diagonal elements are eigenvalues of ${V}_k^{\ast }.$ By step 3, we could obtain *Q*_2 ∣ 1_ which follows ${\chi}_1^2$ distribution, where *p*_2 ∣ 1_ is obtained from ${Q}_{2\mid 1}^{\ast }$.

### MetaQ-test for Meta analysis

Meta-analysis requires summary statistics from each single study. *P* value and z-score have been conventional summary statistics that could combine the results across study, but in GWAS meta-analysis, the methods using *p* value and z-score are generally inferior to the model based meta-analysis; those methods cannot efficiently take account of the between-data-set heterogeneity [17]. In model based meta-analysis, there are two different approaches, fixed effect model and random effect model [18]. Under the fixed effect model, effect sizes of all studies are presumed to be same, in other case, to be different [19]. MetaQ-test considers both cases. Consequently, each QTest has fixed and random versions of meta-analysis.

Another significant feature in MetaQ-test is it is extended keeping the original statistical model structure, structure of QTest. Qtest_1_ is burden type and Qtest_2_ is non-burden type test. This fact is also applied to MetaQ-test. MetaQtest_1_ is a burden and MetaQtest_2_ is a non-burden type test. MetaQ-test keeps not only type of statistics but also the process derived the statistics. By doing that, it can consistently maximize merits of test statistics.

Summary statistics used and the way to synthesize them are essence in meta-analysis [20]. Depending on the type of model-based meat-analysis (fix or random) and the type of test statistics (burden or non-burden), meta-analysis requires different input values and takes different approaches for combining those values.

### Burden test: Meta quadratic Test1 (metaQtest_1_)

#### Meta-analysis assuming homogeneous genetic effects across studies: Homo-meta-Q_1_

First, we extend Qtest1 to meta-analysis maintaining burden type test. We assume that all variants in a gene across studies are from one single study. In Qtest1, to aggregate the effects of variants, ${\hat{\beta}}_{pooled,k}={\alpha}_k^T{W}_k{\hat{\beta}}_k$ is introduced. Similarly, we introduce ${\hat{\beta}}_{pooled}$ that adds up all of variants throughout studies. The way of combining each variant’s effect is exactly same as Qtest_1_, weighted sum of effect sizes of all variants. When we suppose that there are K studies, the estimated pooled effect size of regression coefficients is ${\hat{\beta}}_{pooled}={\alpha}^TW\hat{\beta}={\sum}_{j=1}^m{\alpha}_j{w}_j{\hat{\beta}}_j$, where j is the index for variants, $\hat{\beta}$ is the column vector composed of ${\hat{\beta}}_j$, *α* is the column vector that includes *α*_*j*_ s (j = 1, 2…,m) as components, and W is the diagonal matrix with *w*_*j*_ s as diagonal elements.

$$ {\hat{\beta}}_j={\sum}_{k=1}^K\frac{n_k}{I_1\ast {n}_1+{I}_2\ast {n}_2+\cdots +{I}_K\ast {n}_K}{\hat{\beta}}_{kj},\mathrm{for}\ \mathrm{j}=1,2,\cdots, \mathrm{m}, $$

$$ \hat{\beta}={\left({\hat{\beta}}_1,{\hat{\beta}}_2,\cdots, {\hat{\beta}}_m\right)}^T, $$

$$ {\alpha}_j={\sum}_{k=1}^K{\left(\frac{n_k}{I_1\ast {n}_1+{I}_2\ast {n}_2+\cdots +{I}_K\ast {n}_K}\right)}^2{\upalpha}_{kj},\mathrm{for}\ \mathrm{j}=1,2,\cdots, \mathrm{m}, $$

$$ \alpha ={\left({\alpha}_1,{\alpha}_2,\cdots, {\alpha}_m\right)}^T, $$

$$ {\mathrm{w}}_j={\sum}_{k=1}^K\frac{n_k}{I_1\ast {n}_1+{I}_2\ast {n}_2+\cdots +{I}_K\ast {n}_K}{\mathrm{w}}_{kj},\mathrm{for}\ \mathrm{j}=1,2,\cdots, \mathrm{m}, $$

$$ W=\left[\begin{array}{c}{w}_1\ \\ {}\vdots \\ {}0\end{array}\begin{array}{c}\cdots \\ {}\ddots \\ {}\cdots \end{array}\begin{array}{c}0\\ {}\vdots \\ {}{w}_m\ \end{array}\right]. $$

It is natural that we collapse the variants in the same locus with appropriate weights, such as sample size. Testing hypothesis in this case is *H*_0_ : *β*_*pooled*_ = 0. Since under the null assumption, ${\hat{\beta}}_{pooled}$ is normally distributed with zero mean and variance *α*^*T*^*WVWα*, the Wald type of test statistics is

$$ {Q}_{\mathit{\hom}- meta-q1}={\left(\ {\alpha}^T Wvar\left(\hat{\beta}\right) W\alpha \right)}^{-1}{\hat{\beta}}_{pooled}^2. $$

Since the statistics for meta-analysis is constructed based on the preexisting result of individual level data analysis, $\mathit{\operatorname{var}}\left(\hat{\beta}\right)$ cannot be estimated by the raw data. However, if we assume the independence among studies, we can define $\mathit{\operatorname{var}}\left(\hat{\beta}\right)$ as the block diagonal matrix that has diagonal with $\mathit{\operatorname{var}}\left({\hat{\beta}}_k\right)$,

$$ \mathrm{V}=\operatorname{var}\left(\hat{\beta}\right)=\operatorname{var}\left({\left({\hat{\beta}}_1,{\hat{\beta}}_2,\cdots, {\hat{\beta}}_m\right)}^T\right)=\left[\begin{array}{ccc}{\sum}_{k=1}^K{\left(\frac{n_k}{I_1\ast {n}_1+\cdots +{I}_K\ast {n}_K}\right)}^2\mathit{\operatorname{cov}}\left({\hat{\beta}}_{k1},{\hat{\beta}}_{k1}\right)& \cdots & {\sum}_{k=1}^K{\left(\frac{n_k}{I_1\ast {n}_1+\cdots +{I}_K\ast {n}_K}\right)}^2\mathit{\operatorname{cov}}\left({\hat{\beta}}_{k1},{\hat{\beta}}_{km}\right)\\ {}\vdots & \ddots & \vdots \\ {}{\sum}_{k=1}^K{\left(\frac{n_k}{I_1\ast {n}_1+\cdots +{I}_K\ast {n}_K}\right)}^2\mathit{\operatorname{cov}}\left({\hat{\beta}}_{km},{\hat{\beta}}_{k1}\right)& \cdots & {\sum}_{k=1}^K{\left(\frac{n_k}{I_1\ast {n}_1++\cdots +{I}_K\ast {n}_K}\right)}^2\mathit{\operatorname{cov}}\left({\hat{\beta}}_{km},{\hat{\beta}}_{km}\right)\end{array}\right]. $$

We can easily check that *Q*_*hom* − *meta* − *q*1_ follows chi-square distribution with 1 degree of freedom, because it is the square of single standard normal random variable,$\kern0.5em {\hat{\beta}}_{pooled}$. To draw the statistics, *Q*_*hom* − *meta* − *q*1_, we need estimated ${\hat{\beta}}_{kj}s$ from each study, its variance and MAF weights as inputs. Thus, we call this approach beta-based approach, also because this is burden type, it is like assuming homogeneous effect (same regression coefficients) across the studies.

#### Meta-analysis assuming heterogeneous genetic effects across studies: Het-meta-Q1

Assuming a variant may have a different effect across studies, we can consider the case of heterogeneous effects over studies. This assumption is in accordance with a meta model with random effects. Since we allow the heterogeneity of effect, we can use the results of each study’s regression fit. The model fittings were carried out separately, so the test statistics for association are made of different estimated regression coefficients. Accordingly, the outcome of model fitting in each study that takes account of heterogeneity is appropriate for summary statistics for meta-analysis.

The fact that Q_1_ follows the chi-square distribution also makes us combine Q_1_ s easily. Thus, test statistics of each study would be a naïve and handy summary statistic for meta-analysis

$$ {\mathrm{Q}}_{het- meta-q1}={\sum}_{k=1}^K{Q}_1={\sum}_{k=1}^K{\left({\alpha}_k^T{W}_k\mathit{\operatorname{var}}\left({\hat{\beta}}_k\right){W}_k{\alpha}_k\right)}^{-1}{{\hat{\beta}}_{pooled,k}}^2 $$

However, Q_*het* − *meta* − *q*1_ is a statistic for burden test in respect of sum of single burden statistics. Wald type of statistics for a single variant is distributed as the chi-square distribution with one degrees of freedom. When we assume the independence between studies, then the sum of test statistics also follows a chi-square distribution with K degree freedom, where K is the number of studies in meta-analysis, ${\mathrm{Q}}_{het- meta-q1}\sim {\upchi}_K^2$. In the process of deriving Q_*het* − *meta* − *q*1_, we only require the test statistics. Therefore, we call this approach statistics based meta.

### Non-burden test: Meta quadratic Test2 (metaQtest_2_)

#### Meta-analysis assuming homogeneous genetic effects across studies: Homo-meta-Q2

We extend Qtest2 to meta-analysis in the same manner as the Meta-Qtest1: fixed(homo) and random(hetero) version of meta. However, the main difference between Meta-Qtest1 is that we do not combine the effects of different variants, we dose only collapse the same variants. The basic idea of Meta-Qtest2 is that we regard SNPs on the same locus as the single SNP. For this reason, this approach is different with burden meta-analysis.

It is possible to collapse variants of same locus, because of the assumption of homogeneity in genetic effects size throughout studies. In order to make representative genetic effects size that combines SNPs on the same locus, we use weighted sum. Weights are given as proportional to sample size of study. Representative of regression coefficient of jth SNP is expressed as below,

$$ {\hat{\beta}}_j={\sum}_{k=1}^K\frac{n_k}{I_1\ast {n}_1+{I}_2\ast {n}_2+\cdots +{I}_K\ast {n}_K}{\hat{\beta}}_{kj},\mathrm{for}\ \mathrm{j}=1,2,\cdots, \mathrm{m}. $$

For the sake of simplicity, we assume that the number of variants in a gene is the same across the study, *m* = *m*_1_ = *m*_2_ = ⋯ = *m*_*K*_.

Since all populations do not share the same variants commonly, some variants exit only in a specific population. We thus put an indicator function in front of index of sample size. To maintain the framework of Qtest_2_, we also need variance-covariance matrix of estimated regression coefficients. Under the independence assumption of studies, we derive the variance-covariance matrix, V, analogous to ${\hat{\beta}}_j$ using the sample size proportional weights,

$$ \mathrm{V}=\operatorname{var}\left(\hat{\beta}\right)=\operatorname{var}\left({\left({\hat{\beta}}_1,{\hat{\beta}}_2,\cdots, {\hat{\beta}}_m\right)}^T\right)=\left[\begin{array}{ccc}{\sum}_{k=1}^K{\left(\frac{n_k}{I_1\ast {n}_1+\cdots +{I}_K\ast {n}_K}\right)}^2\mathit{\operatorname{cov}}\left({\hat{\beta}}_{k1},{\hat{\beta}}_{k1}\right)& \cdots & {\sum}_{k=1}^K{\left(\frac{n_k}{I_1\ast {n}_1+\cdots +{I}_K\ast {n}_K}\right)}^2\mathit{\operatorname{cov}}\left({\hat{\beta}}_{k1},{\hat{\beta}}_{km}\right)\\ {}\vdots & \ddots & \vdots \\ {}{\sum}_{k=1}^K{\left(\frac{n_k}{I_1\ast {n}_1+\cdots +{I}_K\ast {n}_K}\right)}^2\mathit{\operatorname{cov}}\left({\hat{\beta}}_{km},{\hat{\beta}}_{k1}\right)& \cdots & {\sum}_{k=1}^K{\left(\frac{n_k}{I_1\ast {n}_1+\cdots +{I}_K\ast {n}_K}\right)}^2\mathit{\operatorname{cov}}\left({\hat{\beta}}_{km},{\hat{\beta}}_{km}\right)\end{array}\right]. $$

The following step is identical to QTest2. Using eigen-decomposed V = *U*Λ*U*^*T*^, we construct the Wald type statistic,

$$ {\mathrm{Q}}_{2- meta}^{Wald}={\hat{\beta}}^T{V}^{-1}\hat{\beta}={\hat{\beta}}^TU{\Lambda}^{-1}{U}^T\hat{\beta}\sim {\upchi}_m^2, $$

$$ \mathrm{where}\kern0.75em \Lambda =\mathit{\operatorname{diag}}\left(\lambda \right). $$

$$ {p}_j=2\left(1-\Phi \right(\frac{u_j^T{\hat{\beta}}_j}{\sqrt{\lambda_j}\left)\right)}, $$

$$ \mathrm{where}\kern0.75em {u}_j={j}^{th}\kern0.5em \mathrm{column}\ \mathrm{of}\ U, $$

$$ {\mathrm{Q}}_{\mathit{\hom}- meta-q2}={\sum}_{j=1}^m{G}_{a,1}^{-1}\left(1-{p}_j\right)\sim 1/2{\upchi}_{2 ma}^2. $$

#### Meta-analysis assuming heterogeneous genetic effects across studies: Het-meta-Q2

For rare variant analysis, overlapped variants with other studies are not many. Thus, the approach of homo-meta-Q2 that aggregates the overlapped SNPs in the same locus could not be appropriate for the rare variant analysis; the estimate of variance-covariance matrix is unstable. An easy alternative way is to assume not homogeneity but heterogeneity of genetic effects. To satisfy this assumption and to accommodate different directional effects among variants in a region, we use the result of single study.

Combining single study results using *p* value is conventional, but this method has a limitation upon achievement of heterogeneity among studies; it just assumes independence among studies. Instead of *p*-value based method, we combine test statistics of each study for Qtest2. Q_2_ follows a mixture of chi-square distributions and the summation of the Q_2_ then also follows a mixture of chi-square distributions.

$$ {\mathrm{Q}}_{het- meta-q2}={\sum}_{k=1}^K{\mathrm{Q}}_2={\sum}_{j=1}^m{G}_{a,1}^{-1}\left(1-{p}_{kj}\right)\sim 1/2\sim 1/2{\upchi}_{2 maK}^2. $$

In Homo/Hetero-meta-Q2, the value of *a* determines the shape of the mixture of chi-square distribution and this value depends on the gene size (number of SNPs in a gene). Therefore, the choice of *a* should be careful.

### Optimal test (unified test): Meta quadratic Test3 (MetaQtest_3_)

We develop an optimal quadratic meta-analysis for robust power gain. A burden type test is known to be more powerful when most variants in a region are causal and their effect directions are the same, but a non-burden type is opposite to this case. Therefore, applying one of the two tests can lose the power to detect the associated variants with a phenotype, if every single gene dose not satisfy the same assumption. A unified approach is to use a weighted average of burden and non-burden test. Since this approach allows two conflicted assumptions about direction among variants (burden or non-burden), an optimal test usually achieves the robust power regardless of directional assumptions.

#### Meta-analysis assuming homogeneous genetic effects across studies: Homo-meta-Q3

When studies are homogeneous, we suggest *Q*_*hom* − *meta* − *q*3_ that is weighted average of *Q*_*hom* − *meta* − *q*1_ and *Q*_*hom* − *meta* − *q*2_. We build a test for meta with an adjustment for homogeneous case. The following are steps for constructing *Q*_*hom* − *meta* − *q*3_. The steps are identical to Qtest3 but for substituting collapsed variables, ($\hat{\beta}$, ${\hat{\beta}}_{pooled}$, *α*, *W*, *V*) instead of vector of variables from each study, (${\hat{\beta}}_k$, ${\hat{\beta}}_{pooled,k}$, *α*_*k*_, *W*_*k*_, *V*_*k*_). The collapsed variables are defined in homo MetaQ-test. Like Qtest3, the empirical distribution of suggested test statistic is calculated from pre-calculated distribution in the step 5.

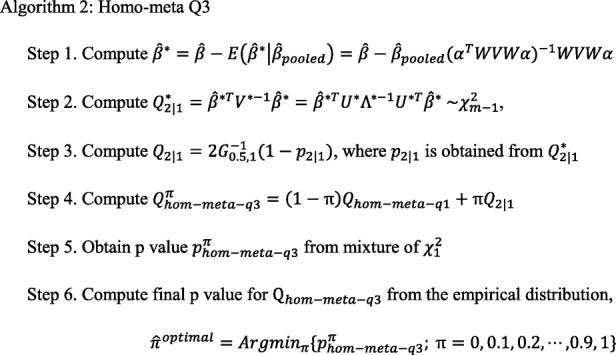



*Meta-analysis Assuming Heterogeneous Genetic Effects across Studies: Het-meta-Q3.*

When studies are heterogeneous, we suggest *Q*_*het* − *meta* − *q*3_ that is weighted average of *Q*_*het* − *meta* − *q*1_ and *Q*_*het* − *meta* − *q*2_. The following are steps for *Q*_*het* − *meta* − *q*3_. The steps are similar to Qtest3 and MetaQTest3 but, in step 2 we use the sum of Wald type statistics, ${Q}_{2\mid 1}^{\ast }$ from each study. For the sake of simplicity, we assume that the number of variants in a gene is the same across the study, thus the degree of freedom is just multiplication K by m.

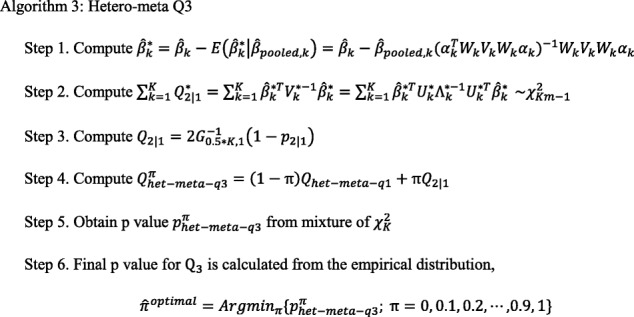



### Numerical simulations

To evaluate the properties of the proposed methods, we performed the simulation study. We generated the genotype data using COSI program that implements coalescent model, and we obtained both length 200 kb and 10,000 European-like haplotypes and African-American-like haplotypes [21]. By randomly mating with haplotypes, we could obtain genotype data for analysis. The 3 kb regions are also randomly selected for each gene and rare variants under the threshold are analyzed [22]. We filtered out common variants with the threshold of MAF larger than 0.03 and singleton SNPs. Left figures consider MAF < 0.05 and right figures consider MAF < 0.01. In European population, 82.93% variants have MAF < 0.01 and 67.51% variants have MAF < 0.001, this frequency is different from that of generated simulation data set in MetaSKAT (86 and 76% respectively). Since COSI program gives randomness in generating haplotypes, this difference could happen even using the same input parameters. Table 1 shows the simulation study settings which we borrowed the basic idea of them in MetaSKAT. For precise comparison of our methods with MetaSKAT, we brought their setting here and tried to prove better performance of MetaQ-test even in the setting for MetaSKAT. Giving variety to kind of population such as sample size and number of covariates, we considered six different scenarios like MetaSKAT. Using COSI program, MetaSKAT generated genotype data set for different populations and created 3 single studies. The number of different scenarios of simulation that they considered is 6, and half of them have the same study sample size and rest of them have different sample size. To give heterogeneity among the studies, in addition to different size, they also allocated different population groups and different number of covariates to each study. We compared the proposed methods with other existing meta-analysis methods. Meta-analysis methods that we considered are as follows: MetaQ-test (Hom-meta-Q1, Het-meta-Q1, Hom-meta-Q2, Het-meta-Q2, Hom-meta-Q3, Het-meta-Q3), MetaSKAT (Hom-meta-SKAT, Het-meta-SKAT, Hom-meta-SKAT-O, Het-meta-SKAT-O), Meta-Burden, Fisher’s combined probability test and Stouffer’s Z-score method [23, 24].

**Table 1:** Simulation study settings

Scenario	Population	Sample Size			Covariates		
		Study 1	Study 2	Study 3	Study 1	Study 2	Study 3
1	EUR	1600	2200	3200	(x_1_, x_2_)	(x_1_, x_2_)	(x_1_,x_2_)
2	EUR	1600	2200	3200	(x_1_)	(x_1_, x_2_)	(x_1_,x_2_,x_3_)
3	EUR + AA	1600	2200	3200	(x_1_)	(x_1_, x_2_)	(x_1_,x_2_,x_3_)
4	EUR	2400	2400	2400	(x_1_, x_2_)	(x_1_, x_2_)	(x_1_,x_2_)
5	EUR	2400	2400	2400	(x_1_)	(x_1_, x_2_)	(x_1_,x_2_,x_3_)
6	EUR + AA	2400	2400	2400	(x_1_)	(x_1_, x_2_)	(x_1_,x_2_,x_3_)

EUR + AA denotes that population of first and second study is European and the population of Study3 is African-American

#### Type I error and power simulations

For type I error simulations, we generated 100 phenotypes for 100 each gene under the null hypothesis; there is no association between a gene and a phenotype.

2 $$ {y}_{ki}=0.5{X}_{k1i}+\cdots +0.5{X}_{k{q}_ki}+{\varepsilon}_{ki},\kern1.5em {\varepsilon}_{ki}\sim N\left(0,1\right), $$

As the type I error simulation of MetaSKAT, *X*_*k*1*i*_ is the covariate taking 0 or 1 value with equal probability 0.5. The rest of covariates, from *X*_*k*2*i*_ to ${X}_{k{q}_ki}$ are taken from a standard normal distribution. The information of covariates for each study is in the Table 1. The index, *q*_*k*_ indicates the number of covariates for kth study. Since we generated 100 genes and 100 phenotypes, we carried out 10,000 times association tests, and the level of significance, we gave α = 0.01 and 0.001.

Unlike type I error simulations, we generated 1000 genotype datasets and made phenotypes using the below model for power simulations,

3 $$ {y}_{ki}=0.5{X}_{k1i}+\cdots +0.5{X}_{k{q}_ki}+{\boldsymbol{G}}_{\boldsymbol{k}\boldsymbol{i},\boldsymbol{causal}}^{\prime }{\boldsymbol{\beta}}_{\boldsymbol{k},\boldsymbol{causal}}+{\varepsilon}_{ki},\kern1.5em {\varepsilon}_{ki}\sim N\left(0,1\right) $$

***G***_***ki***, ***causal***_ is a vector of causal variants in a gene, and ***β***_***k***, ***causal***_ is their effect size. For the proportion of causal variants, we set four cases, 10, 20, 30 and 50% of variants are causal like in MetaSKAT. To illustrate the effect of burden or non-burden type test, we also assumed that all variants are positive or 80% are positive and rest of 20% are negative. Since the number of causal variants or positive variants could not be integer in some cases because of small number of variants in a gene, we generated number of causal variants in Bernoulli distribution. The regression coefficient of genetic effect is given same as the MetaSKAT, ***β***_***k***, ***causal***_ = c|*log*_10_(*MAF*)|. However, we used study specific MAF rather than population MAF, because in reality we hardly get the population MAF even in Meta-analysis. Defining the coefficient in this way reflects the assumption of rare variant study; the rarer SNPs have the larger effects on the phenotype. We set c = 0.475 in 5% of causal variants, 0.375 for 10%, 0.25 for 20%, and 0.175 for 50% of causal variants respectively. Since the effect size of regression coefficient depends on the MAF, the case for different MAF across the studies assumes heterogeneous effect and the case for the same MAF assumes homogeneous effect. These assumptions rely on the belief that different populations could have different MAF, and this phenomenon is called population stratification. In the simulation work, there are some differences with MetaSKAT. First, we used haplotypes that have different distribution of allele frequencies with that of MetaSKAT and second, we used study specific MAF rather than population MAF that was used in simulation of MetaSKAT. Finally, we used Bernoulli distribution to assign causal variants rather than exact given proportion percentages. We expected that the differences might cause the slightly different power results of MetaSKAT calculated here with MetaSKAT paper.

## Materials

### Meta-analysis for gene-level rare variants association studies

We analyzed whole exome sequencing data from Type 2 Diabetes Genetic Exploration by Next-generation sequencing in multi-Ethnic Samples (T2D-GENES) consortium. Sequencing was performed at the Broad Institute with Agilent v2 capture reagent on HiSeq platform. The consortia have exome sequencing data of 13,000 individuals from 5 different ancestry groups: African Americans (AJ, AW), American Hispanics (HA, HS), East Asian (EK, ES), South Asians (SL, SS), and European (UA, UF, UG, US, and UB). The words in parenthesis stand for the populations in the ancestry group. For meta-analysis, we selected East Asian ancestry group, EK from Korean samples and ES from Singaporean samples. Total number of samples in EK is 1086 and in ES is 1078. The primary goal of two consortia is to identify novel type 2 diabetes (T2D) related genetic factors, but data also includes several diabetes-related quantitative traits such as lipid and blood pressure traits. We applied the proposed meta methods to lipid and blood pressure traits: total cholesterol (CHOL), HDL cholesterol (HDL), LDL cholesterol (LDL), triglycerides (TG), systolic blood pressure (SBP) and diastolic blood pressure (DBP). Subjects having medication that might affect the traits were excluded for the analysis. Sample sizes considered missing count and medication are described in Table 2. For covariates, we used age, sex information (BMI information was used only for blood pressure phenotypes).

**Table 2:** Sample Size of Asian Population Groups for Seven Quantitative Traits

Traits	CHOL	HDL	LDL	TG	SBP	DBP
EK (1086)	1078	1078	1031	1078	1086	1086
ES (1078)	627	628	621	627	1077	1077

Missing samples and samples who receive medication are excluded

## Results

### Type I error

Empirical type I error rates under the setting of Scenario 1 and 2 are calculated. Since first scenario consider homogeneous case and second scenario considers heterogeneous case, we only calculate type I error in these two scenarios. The rates are calculated under each significant level, 0.01 and 0.001. Empirical type I error rates under the setting of Scenario 1 are given in Table 3. For both MetaQTest and MetaSKAT, type I error rates were well controlled, but in Het-meta-SKAT-O, type I error rate was somewhat inflated. Empirical type I error rates under the setting of Scenario 2 are given in Table 4. For both MetaQ and MetaSKAT, type I error rates were also well controlled, but in Hom-meta-Q2, type I error rate was somewhat inflated.

**Table 3:** Type I Error Rates Estimates at in Scenario 1

α	Hom-meta-Q1	Het-meta-Q1	Hom-meta-Q2	Het-meta-Q2	Hom-meta-Q3	Het-meta-Q3	Hom-meta-SKAT	Het-meta-SKAT	Hom-meta-SKAT-O	Het-meta-SKAT-O
10^−2^	9.30E-03	9.00E-03	1.13E-02	1.26E-02	1.13E-02	1.21E-02	1.51E-02	1.05E-02	1.38E-02	1.23E-02
10^−3^	1.30E-03	1.10E-03	1.10E-03	1.30E-03	1.30E-03	9.00E-04	1.60E-03	1.00E-03	2.10E-03	1.80E-03

**Table 4:** Type I Error Rates Estimates at in Scenario 2

α	Hom-meta-Q1	Het-meta-Q1	Hom-meta-Q2	Het-meta-Q2	Hom-meta-Q3	Het-meta-Q3	Hom-meta-SKAT	Het-meta-SKAT	Hom-meta-SKAT-O	Het-meta-SKAT-O
10^−2^	1.08E-02	9.90E-03	1.05E-02	9.80E-03	9.80E-03	1.00E-02	9.80E-03	1.03E-02	9.80E-03	1.04E-02
10^−3^	1.40E-03	9.00E-04	1.40E-03	1.10E-03	9.00E-04	1.10E-03	1.00E-03	9.00E-04	1.10E-03	1.20E-03

### Power

**Fig. 1:**
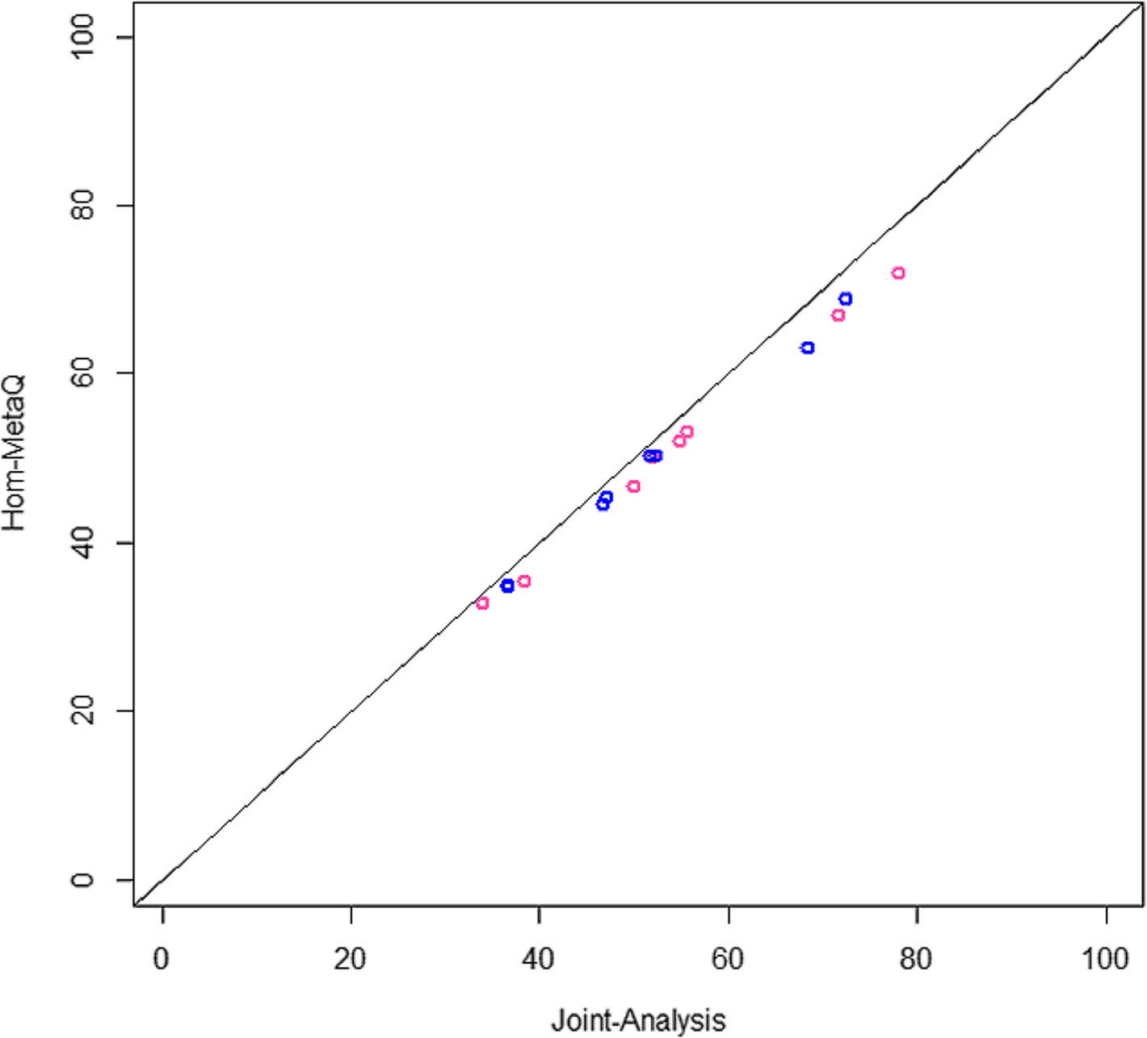
Power comparisons between joint-analysis and Hom-MetaQ. Blue dots indicate power percentages in 8 cases of scenario 1 and pink dots for scenario 4

To compare the power, we performed the simulation work. First, we compared power of our meta method with those of joint analysis to check efficiency of meta method. When meta-analysis yields highly comparable results with joint analysis, the meta method serves well as an alternative of joint analysis. Since joint-analysis cannot handle heterogeneity between studies such as different number of covariates directly, we only performed joint-analysis in homogeneous scenario settings (scenario 1 and 4). In Fig. 1, the blue 8 dots indicate each case for senario1 (causal 5 to 50% and all same direction of variants to different direction) and the pink dots are for scenario 4. Joint-analysis is consistently powerful, but Hom-meta-Q also achieves highly comparable power compared with joint-analysis. Especially, for scenario 1, our meta methods recovers almost power of joint-analysis.

**Fig. 2:**
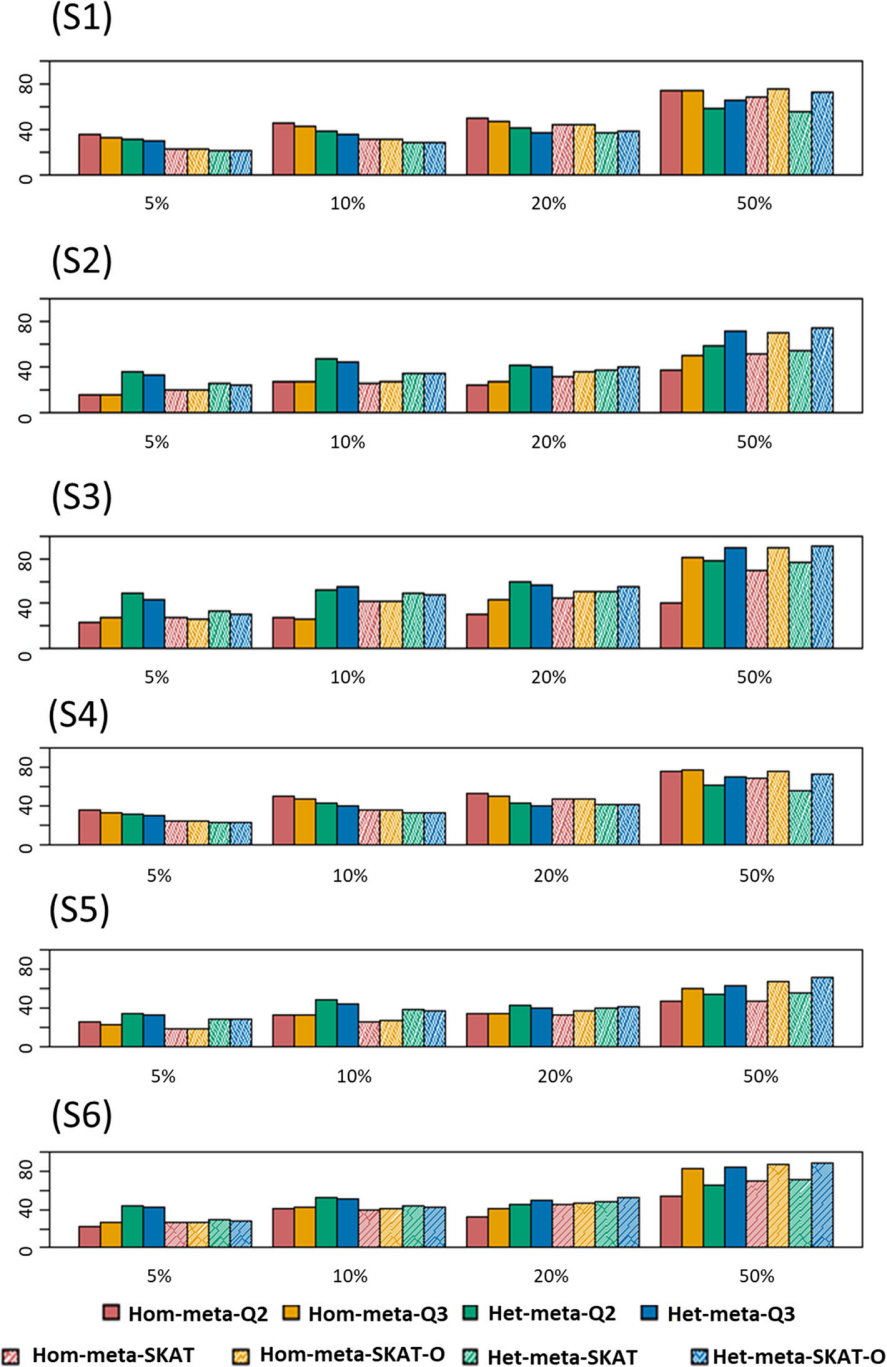
Power comparisons of the eight meta methods when all Causal Variants in a Region are Deleterious

Figure 2 shows power comparisons of eight different meta methods when all causal variants are risk increasing. In the figure, first 4 bars indicate our proposed methods, Hom-meta-Q2, Hom-meta-Q3, Het-meta-Q2 and Het-meta-Q3 in order. The next 4 bars indicate competing MetaSKAT methods, Hom-meta-SKAT, Hom-meta-SKAT-O, Het-meta-SKAT and Het-meta-SKAT-O. In the first scenarios, since we assume homogeneous genetic effects, Hom-meta-Q are more powerful than Het-meta-Q. Up to 20% causal, Hom-meta-Q2 is the most powerful but in 50% causal case, as the power of burden test increases, Hom-meta-Q3, optimal type, becomes the most powerful. In these scenarios, our methods outperform in 5%~ 20% percentages of causal variants. However, when a causal percentage reaches 50%, then MetaSKAT (Hom-meta-SKAT-O) overtakes the power (Hom-meta-Q3: 74.2% and Hom-meta-SKAT-O: 76.4%). The reversal occurs, because Hom-meta-Q1 works poorly even Hom-meta-Q2 is more powerful than Hom-meta-SKAT when it is expected to outperform in a case of high causal percentage. But, the power differences are not so big, about 2% difference. Inscenario 2, we gave heterogeneity between studies by giving different number of covariates. The powers of Het-meta-Q are higher than those of Hom-meta-Q and their power differences are clearly different, about twice higher in 5% of causal variants. Het-meta-Q outperform except in the case of 50% of casual variants and this trend also appears in the rest of scenarios: as the number of causal variants increases, MetaSKAT is more powerful than our method. The reason why this trend is maintained is that our burden type test has lower power than Meta-Burden and even our non-burden type test when burden type test should have the highest power is more powerful. In scenario 3, population of studies is different and the number of covariate is also different. In this more heterogeneous case, Het-meta-Q has higher power than Hom-meta-Q, and the value of power itself is higher than in scenario 2. Our method outperform up to 20% of causal variants. As the percentage of casual variants increases the power of our method becomes lower than MetaSKAT. One of the distinguishable features of our method is that the power of our methods varies sensitively depending on the range of heterogeneity. The gap of power differences of homo and heterogeneous cases are larger than those of MetaSKAT. All methods in MetaSKAT have the robust power but in 50% of casual variants. Thus, when there are information about heterogeneity prior to analysis and the percentage of causal variants is expected to be lower, then our method is more powerful than MetaSKAT. For the rest of scenarios, we gained similar power results with previous scenarios; only in the case of 50% causal variants, MetaSKAT has higher power than MetaQ, but in scenario 4, our methods outperformed regardless of causal variants percentages and in scenario 6, in the case of 20% causal variants with 50% causal, MetaSKAT outperforms.

**Fig. 3:**
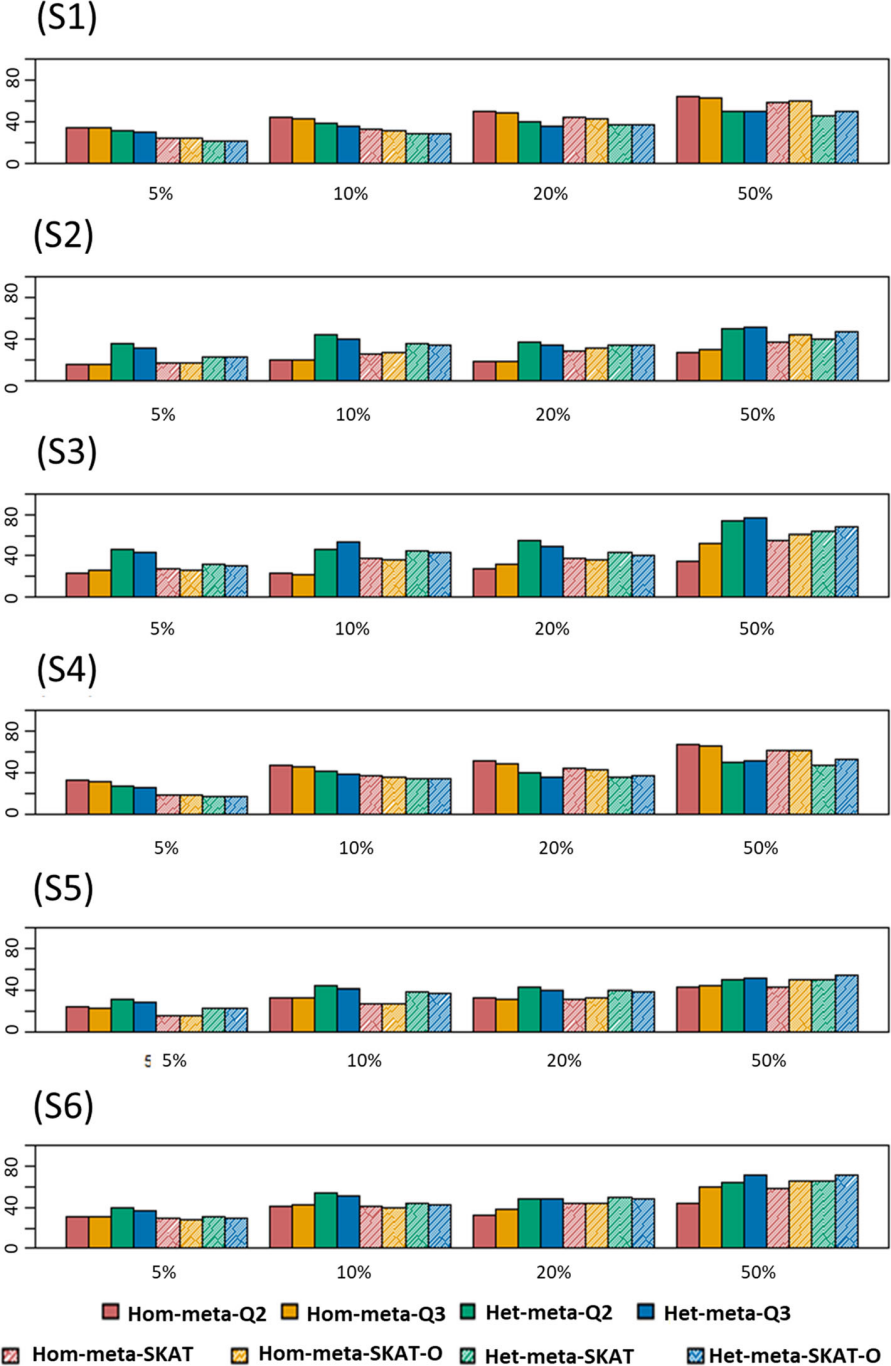
Power comparisons of the eight meta methods when 20% causal variants in a region are protectious and 80% are deleterious

Figure 3 shows power comparisons of meta methods when 20% of causal variants are risk decreasing and 80% are risk increasing. In scenario 1, Hom-meta-Q have higher power than MetaSKAT regardless of percentage of causal variants. However, the power differences become smaller as casual percentage increases. Compared to the case of all risk causal variants, in 50% of causal variants our method still have higher power because power loss of burden type is not large. In scenario 2 and 3, Het-meta-Q have higher power than MetaSKAT. Our methods show significant difference between Hom-meta-Q and Het-meta-Q, but MetaSKAT has little difference between their methods. In scenario 5, up to 20% causal, our best method is powerful than MetaSKAT, but when causal variants are 50%, then MetaSKAT outperforms. Moreover, in scenario 6, in only 20% causal, MetaSKAT outperforms, but the power differences between our proposed methods and MetaSKAT are smaller than those observed in the previous, because in this setting non-burden type test is more powerful than burden, so our burden type test that is less powerful is less involved here. The reason why our burden type test, Hom-meta-Q, have little power in heterogeneous case is that the assumption used in burden type test is not kept well in heterogeneous case. In heterogeneous case, we can hardly say that all of variants in a gene across the different population groups are detected in any population and have same direction. Thus, we do not include the result of Hom-meta-Q in simulation and real data analysis.

### Real data analysis results

We applied suggested methods to the real lipid and blood pressure data. All considered meta methods did not detect any significant genes for lipid traits. But, for blood pressure traits we gained the different association results with lipid traits. Before analysis, we filtered common SNPs. Thus, remaining number of genes for the analysis of SBP is 9303 and SNPs in each gene have MAF < 0.05 and MAC > 4. Unlike MetaSKAT methods that failed to detect any significant genes as well in the lipid traits analysis (at the bonferroni’s significant level and arbitrary level, 1.00e-04), MetaQ-test detected PCDHA9 gene in Het-meta-Q2 (*p* value = 4.75e-05) at the bonferroni’s significant level (*α* = 5.37e-06), but this gene is also not known to be related with blood pressure. According to GO annotations, it is involved in calcium ion binding. However, the gene that has the second smallest *p* value in both Het-meta-Q2 (*p* value = 1.19e-05) and Het-meta-Q3 (*p* value = 2.41e-05) is KCNA5 and this gene is already known for having strong relevance to hypertension [25, 26]. Table 5 shows that *p* values of genes that were detected at the threshold, 1.00e-04 have the smallest value in MetaQ-test. Moreover, we compared our meta method results with that of single QTest analysis to verify the improved power of meta methods. Table 6 shows that PCDHA9 gene is also detected in QTest2 in EK single study analysis and its *p* value (*p* value = 8.22e-07) is smaller than in Het-meta-Q2. However, its *p* value in ES single study analysis has very small value, so *p* value from MetaQ-test is the compromised these two *p* values from EK and ES single QTest analysis. But, *p* values of KCNA5 from each single QTest are both larger than *p* value from MetaQ-test. This fact can say that KCNA5 which is known for one of causal genes of hypertension arterial was only detected by MetaQ-test and MetaQ-test improved the statistical power of QTest.

**Table 5:** Meta-Analysis Results for Testing the Rare Variants Effects on Systolic Blood Pressure

Meta-Analysis		GENE	
Method	Test type	PCDHA9 (CHR 5)	KCNA5 (CHR 12)
meta Q tests	Het-meta-Q1	6.18E-01	1.03E-01
	Het-meta-Q2	**4.75E-06**	**1.19E-05**
	Het-meta-Q3	7.34E-06	2.41E-05
meta SKAT	Hom-meta-SKAT	3.93E-03	5.03E-04
	Het-meta-SKAT	3.72E-03	1.68E-04
	Hom-meta-SKAT-O	7.65E-03	1.13E-03
	Het-meta-SKAT-O	7.60E-03	4.67E-04
Other methods	Meta-burden	7.97E-01	2.59E-01
	Fisher’s method (Q3)	8.18E-06	3.06E-05
	Score method (Q3)	2.23E-04	1.31E-05

Bonferroni corrected significant level for Meta-analysis is *α* = 6.82e-06

**Table 6:** Single QTest Analysis Results for Testing the Rare Variants Effects on Systolic Blood Pressure

Single QTest		GENE	
Population	Test type	PCDHA9 (CHR 5)	KCNA5 (CHR 12)
EK	EK-Q1	6.82E-01	6.66E-01
	EK-Q2	**8.22E-07**	1.83E-03
	EK-Q3	**1.33E-06**	1.53E-03
ES	ES-Q1	3.73E-01	3.68E-02
	ES-Q2	2.48E-01	4.69E-04
	ES-Q3	3.97E-01	1.43E-03

EK GWAS significant level is *α* = 6.30e-06 and ES GWAS significant level is *α* = 6.82e-06

Bonferroni corrected significant level for Meta-analysis is *α* = 6.82e-06

For the analysis of DBP, after filtering common variants, the number of genes is 10,528 and SNPs in each gene have MAF < 0.05 and MAC > 3. In the meta-analysis of DBP, two genes, DTYMK and PCCA were detected at the Bonferroni’s significant level (*α* = 4.75e-06). Table 7 shows that Meta-Analysis results for testing the rare variants effects on DBP. DTYMK was detected in Het-meta-Q3 (*p* value = 1.17e-06) and PCCA was detected in Hom-meta-SKAT-O (*p* value = 3.18e-06). DTYMK, however, is only found in EK samples, thus MetaQ-test result is only stemmed from EK QTest result (Table 8 shows that *p* values of QTest are the same with that of MetaQ-test). When a gene exists in a single study, then our MetaQ-test result reflects the result of single study. DTYMK is not currently identified as related with blood pressure, but as related with thymidylate kinase activity. Another detected gene in MetaSKAT, PCCA is not known to be associated with blood pressure function, but it is related with propionic academia and pcca-related propionic academia.

**Table 7:** Meta-Analysis Results for Testing the Rare Variants Effects on Diastolic Blood Pressure

Meta-Analysis		GENE		
Method	Test type	DTYMK (CHR 2)	CABIN1 (CHR 22)	PCCA (CHR 13)
meta Q tests	Het-meta-Q1	8.87E-01	5.95E-01	1.59E-01
	Het-meta-Q2	1.21E-06	8.67E-06	1.92E-04
	Het-meta-Q3	**1.17E-06**	**6.91E-06**	7.92E-05
meta SKAT	Hom-meta-SKAT	1.06E-01	3.93E-03	8.63E-06
	Het-meta-SKAT	1.43E-02	4.97E-03	8.11E-06
	Hom-meta-SKAT-O	1.40E-01	7.74E-03-	**3.18E-06**
	Het-meta-SKAT-O	2.42E-02	1.03E-02	3.76E-06
Other methods	Meta-burden	1.51E-01	5.09E-01	4.78E-01
	Fisher’s method (Q3)	NA	1.65E-05	1.05E-04
	Score method (Q3)	NA	1.50E-05	1.33E-02

GWAS significant level is *α* = 4.75E-06

The minimum *p* value in each gene among the meta methods is marked in bold

Fisher’s method and Z-score method used *p* values of optimal version of QTest, QTest3

NA denotes that *p* value is not calculated because of very low valued statistics

**Table 8:** Single QTest Analysis Results for Testing the Rare Variants Effects on Diastolic Blood Pressure

Single QTest		GENE		
Population	Test type	DTYMK (CHR 2)	CABIN1 (CHR 22)	PCCA (CHR 13)
EK	EK-Q1	8.87E-01	7.84E-01	7.28E-01
	EK-Q2	1.21E-06	2.22E-02	7.84E-01
	EK-Q3	**1.17E-06**	2.33E-02	8.72E-01
ES	ES-Q1	NA	3.26E-01	**2.76E-06**
	ES-Q2	NA	3.00E-05	1.52E-05
	ES-Q3	NA	4.81E-05	9.45E-06

Bonferroni corrected significant level for Meta-analysis is *α* = 6.82e-06

PCCA gene was also detected using QTest1 of ES sample analysis (*p* value = 2.76e-06, Table 8). But EK QTest results offset the results of MetaQ-test, so MetaQ-test could not detect this gene.

Although CABIN1 gene was not discovered at the Bonferroni’s significant level, but it is discovered at the FDR adjusted *p* value. CABIN1 which was detected using Het-meta-Q2 and Het-meta-Q3 (*p* value = 8.67e-06 and 6.91e-06 respectively) is known to be associated hypertension arterial and purpura thrombotic thrombocytopenic that is closely related blood pressure. Table 8 shows that QTest results of EK and ES population groups. CABIN1 was not detected using single QTest.

## Discussion and conclusion

We propose MetaQ-test for meta-analysis of gene-level rare variant association studies. The basis of MetaQ-test is preserving the prior phase of association studies that is Q-test. MetaQ-test retains the relationship among multiple variants in a region. Further, MetaQ-test considers whether the genetic effects on the phenotype are same across studies or not. Assuming same effects corresponds to fixed effect meta-analysis model or else random effect model. By considering direction of variant effects and their equivalence in effect size at the same time, MetaQ-test can cover broad range of realistic meta-analysis cases.

We investigated the performance of MetaQ-test through simulation and real data analysis. Simulation studies showed that type I error rates were controlled well and MetaQ-test, particularly Het-meta-Q achieved the higher than or as powerful as MetaSKAT in various scenarios. However, when causal variants are over than 50%, then our methods are not powerful as MetaSKAT. Thus, when there are small percentage of causal variants, our method is more powerful in all scenario settings. Since Hom-meta test uses estimating regression coefficients, satisfaction of model assumption affects the power of MetaQ-test sensitively. If there are no many overlapped variants in a gene across single studies, the assumption of Hom-meta is broken and Hom-meta-Q hardly perform well. For this reason, in the result of real data analysis (EK and ES sample have the small proportion of shared SNPs in a gene), Hom-meta-Q have inflated *p* values and we thought that most of them are false positive. Thus, in the result of real data analysis, we excluded the Hom-meta-Q results. Therefore, we expect that the prior test of heterogeneity of genetic effects can help us to determine appropriate model. In real data analysis, we have shown that Het-meta-Q searched out some known genes associated blood pressure trait. However, it also discovered some novel genes that are not at known to be associated with blood pressure at least for now, thus to validate biological relationship between them, more research and experiment in biology field are needed. For the future research, we can combine adaptive test that can determine degree of heterogeneity of genetic effects to improve the power further.

## Abbreviations

AJ: African American Jackson Heart Study Candidate Gene Association Resource
AW: African American Wake Forest Study
BMI: Body mass index
CHOL: Cholestrol
DBP: Diastolic blood pressure
EK: East Asian Korea Association Research Project (KARE) and Korean National Institute of Health (KNIH)
ES: East Asian Singapore Diabetes Cohort Study and Singapore Prospective Study Program
GWAS: Genome wide association test
HA: Hispanic San Antonio Mexican American Family Studies, Texas
HDL: High-density lipoprotein cholestrol
HS: Hispanic Starr County, Texas
LDL: Low-density lipoprotein cholestrol
QTest: Quadratic test
SBP: Systolic blood pressure
SKAT: Sequence kerneal association test
SL: South Asian London Life Sciences Population (LOLIPOP)
SS: South Asian Singapore Indian Eye Study
T2D-GENES: A consortium for Type 2 Diabetes Genetic Exploration by Nexte-generation sequencing in multi-Ethnic Samples
TG: Triglycerides
UA: European Longevity Genes in Founder Populations (Ashkenazi)
UB: European Malmo-Botnia Study
UF: European Metabolic Syndrome in Men Study (Finnish)
UG: European Kooperative Gesundheitsforschung in der Region Augsburg (KORA)
US: UK Type 2 Diabetes Genetics Consoritum
